# Exploring the Contribution of Curcumin to Cancer Therapy: A Systematic Review of Randomized Controlled Trials

**DOI:** 10.3390/pharmaceutics15041275

**Published:** 2023-04-19

**Authors:** Chiara de Waure, Carlotta Bertola, Gaia Baccarini, Manuela Chiavarini, Cesare Mancuso

**Affiliations:** 1 Department of Medicine and Surgery, University of Perugia, Piazza L. Severi 1, 06132 Perugia, Italy; chiara.dewaure@unipg.it (C.d.W.); carlotta.bertola@studenti.unipg.it (C.B.); gaia.baccarini@studenti.unipg.it (G.B.); manuela.chiavarini@unipg.it (M.C.); 2 Fondazione Policlinico Universitario A. Gemelli IRCCS, Largo F. Vito 1, 00168 Rome, Italy; 3 Department of Healthcare Surveillance and Bioethics, Section of Pharmacology, Università Cattolica del Sacro Cuore, Largo F. Vito 1, 00168 Rome, Italy

**Keywords:** chemotherapy, nutraceuticals, radiotherapy, tumor, turmeric

## Abstract

Although the anticancer role of curcumin has been extensively addressed in preclinical research, only a few studies were carried out in humans, with conflicting results. The aim of this systematic review is to collate together the results of the therapeutic effect of curcumin in cancer patients. A literature search was carried out in Pubmed, Scopus, and the Cochrane Central Register of Controlled Trials up to 29 January 2023. Only randomized controlled trials (RCTs) designed to evaluate the effects of curcumin on cancer progression, patient survival, or surgical/histological response were included. Seven out of 114 articles, published between 2016 and 2022, were analyzed. They evaluated patients with locally advanced and/or metastatic prostate, colorectal, and breast cancers, as well as multiple myeloma and oral leucoplakia. Curcumin was given as an add-on therapy in five studies. Cancer response was the most investigated primary endpoint and curcumin issued some positive results. On the contrary, curcumin was ineffective in improving overall or progression-free survival. The curcumin safety profile was favorable. In conclusion, available clinical evidence is not strong enough to support the therapeutic use of curcumin in cancer. New RCTs exploring the effects of different curcumin formulations in early-stage cancers would be welcome.

## 1. Introduction

Curcumin (1,7-bis[4-hydroxy 3-methoxy phenyl]-1,6-heptadiene-3,5-dione) is a polyphenol extracted from the rhizome of *Curcuma longa Linn* (family *Zingiberaceae*). It is commonly known as turmeric and is extensively used in the Asian continent to make food colored and flavored [1,2]. In addition to culinary use, traditional Indian medicine considers turmeric an effective remedy in the treatment of several diseases [2,3,4]. Together with curcumin, which is the most abundant polyphenol (~77%), the rhizome of *Curcuma longa* also contains other phenol-based compounds called curcuminoids, the most important being desmethoxycurcumin (~15%) and bisdemethoxycurcumin (~3%) [3,4]. Considering that curcumin prevails over the other congeners, most of the preclinical literature in this field has explored the effects of the pure compound in several experimental systems, whereas only a few papers have studied the biological properties of curcuminoids [5,6,7,8,9]. Unlike preclinical research, which suggested a beneficial role for curcumin in neurodegenerative, cardiovascular, hematological, and infectious diseases, only a few studies have been carried out to confirm these therapeutic effects in humans [10,11,12]. A plausible reason for this underestimation is that curcumin has unfavorable pharmacokinetics, characterized by poor bioavailability after oral administration and negligible plasma and tissue levels [2]. Therefore, with the purpose to improve absorption, distribution, and tissue accumulation, novel oral formulations of curcumin have been prepared, including either an extract enriched with curcuminoids and sesquiterpenoid components of turmeric (BCM-95 CG) or complexes with liposoluble vehicles, such as phospholipids or nanoparticles (Table 1). A careful analysis of Table 1 shows that either the presence of sesquiterpenoids (45% Ar-turmerone) or the complexation with phospholipids (~40% soy lecithin and ~40% microcrystalline cellulose) and nanoparticles (containing 46% glycerin, 4% gum ghatti, and 38% water) markedly increases the curcumin peak plasma concentration (C_max_), suggesting a more effective absorption of the active ingredient. Simultaneously, the increase in the area under the curve (AUC_0–24h_) demonstrates how the presence of either sesquiterpenoids or nanoparticles is capable of improving the bioavailability of curcumin. Lastly, the increase in half-life (T_1/2_) implies an extension of the time of persistence of curcumin in the body and, therefore, a more prolonged pharmacological action. The composition of these formulations is provided in Table 1.

With regard to pharmacodynamics, preclinical studies have shown that curcumin reduces free radical- or copper-induced lipid peroxidation in several experimental systems [18,19]. Furthermore, structure activity studies demonstrated the importance of the β-diketone moiety and phenolic hydroxyl group for cytoprotective activity [18,19]. Together with this direct antioxidant effect, curcumin has been shown to regulate several intracellular systems, such as the transcription factors nuclear factor kB (NFkB) and vascular endothelial growth factor (VEGF), the kinases phosphoinositide-3 kinase/Akt (PI3K/Akt) and cyclin-dependent kinase (cdk), the proinflammatory interleukins (IL) IL-1β, IL-6, and IL-23, and many other proteins involved in apoptosis (Bax and Bcl-2) and cell stress response (heme oxygenase-1 and heat shock-protein-70) [3,11,20,21]. This wide array of interactions, in particular those with genes/proteins involved in cell proliferation/survival and angiogenesis, prompted investigators to explore the therapeutic role of curcumin in cancer. In this context, thousands of articles demonstrated that curcumin, alone or in combination with chemotherapeutic agents, is able to counteract cell proliferation, invasion, and metastatic potential through the regulation of specific targets or epigenetic mechanisms [20,21]. Unfortunately, the vast majority of these studies were carried out on laboratory animals or cell lines, using curcumin at concentrations several orders of magnitude greater than those achieved in human plasma and tissues, thus limiting the translational interest of these studies. Conversely, only a handful of clinical trials investigated the anticancer effects of curcumin, and only a fraction of these studies were focused on its impact on cancer progression and/or patient survival, with conflicting results. Nevertheless, the interest of the scientific community in the efficacy of this nutraceutical in cancer is still alive thanks to clinical evidence that corroborates the beneficial role of curcumin in improving the quality of life of cancer patients and preventing radiotherapy-induced adverse effects. In this regard, several systematic reviews and meta-analyses, supporting the beneficial role of curcumin in counteracting radiation-induced mucositis or skin lesions, are available in the literature [22,23,24,25].

In this context, the aim of this paper is to provide a systematic review of the articles that assessed the therapeutic effects of curcumin in cancer patients. With the purpose of providing an original contribution to the field, this analysis has been narrowed to randomized controlled trials (RCTs) in which hard endpoints were evaluated. In particular, the attention was focused on RCTs designed to evaluate the effects of curcumin, alone or as an add-on therapy, on cancer progression, patient survival, or surgical/histological response.

## 2. Materials and Methods

This systematic review was conducted and reported according to the preferred reporting items for systematic reviews and meta-analyses (PRISMA) [26].

### 2.1. Search Strategy

A literature search restricted to RCTs was carried out on Pubmed, Scopus, and the Cochrane Central Register of Controlled Trials from the databases’ inception up to 29 January 2023. The following keywords were used to perform the literature search in Pubmed: ((curcumin[Title] OR turmeric[Title]) AND (cancer*[Title] OR tumor*[Title])). The same keywords, always searched in the title field, were used for the literature search in both Scopus and the Cochrane Center Register of Controlled Trials. No other restraints were applied.

### 2.2. Study Selection

A two-step approach was used to select eligible articles after the removal of duplicate publications. First, articles were screened based on titles and abstracts, and then full texts of potential eligible papers were obtained and checked for final inclusion. For each potentially included study, two investigators independently conducted the selection and data abstraction. Disagreements were resolved by discussion or consultation with a third author. Articles were considered eligible if they reported the results of RCTs with parallel arm design and aimed to explore the use of curcumin—alone or as an add-on to other anticancer therapies—in any dosage and formulation in patients with any cancer in improving the clinical response, in terms of the following hard endpoints, studied as either primary or secondary outcomes: overall survival (OS), progression-free survival (PFS), objective response, time to tumor progression (TTP), and duration of off treatment. The following studies were excluded: single-arm or open-label RCTs, including those designed to address the pharmacokinetics of curcumin; RCTs whose outcomes were assessed through soft endpoints or related to local effects of curcumin on mucositis, radiodermatitis and other skin lesions; RCTs analyzing the effects of curcumin in combination with other nutraceuticals (e.g., piperine to improve bioavailability or resveratrol to increase the cytoprotective effect); study protocols, conference abstracts, and reviews; and publications describing the therapeutic effects of curcumin on diseases different than cancer.

### 2.3. Quality Assessment

The quality of included RCTs was assessed by the Cochrane Risk of Bias Tool (RoB-Tool 2) [27] by two researchers independently, and any disagreement was solved with the involvement of a third researcher. The RoB-Tool 2 allows issuing a judgment on the risk of bias (low, some concerns, high) with respect to the following domains: randomization process, deviations from intended interventions, missing outcome data, measurement of the outcome, and selection of the reported results. The overall risk of bias is then considered low, if all domains are judged to be at a low risk, or high, if at least one domain is judged to be at a high risk of bias or multiple domains are judged to have some concerns in a way that substantially lowers the confidence of the results. The results of the quality assessment were reported in a descriptive way.

### 2.4. Data Extraction and Synthesis

A data extraction form was used to gather information on the following aspects: first author’s last name, year of publication, country, trial design and duration, study population characteristics (type and stage of cancer, previous treatment, gender, age), number of participants in the experimental and control arms, type of intervention (dosage and formulation of curcumin), type of control, and study endpoints and their results. This information was collected by one researcher and checked by a second one. A narrative synthesis of the results was planned in the light of expected heterogeneity in terms of cancer patients, curcumin dosage and formulation, and time of the assessment of the study endpoints.

## 3. Results

### 3.1. Study Search and Selection

**Figure 1:**
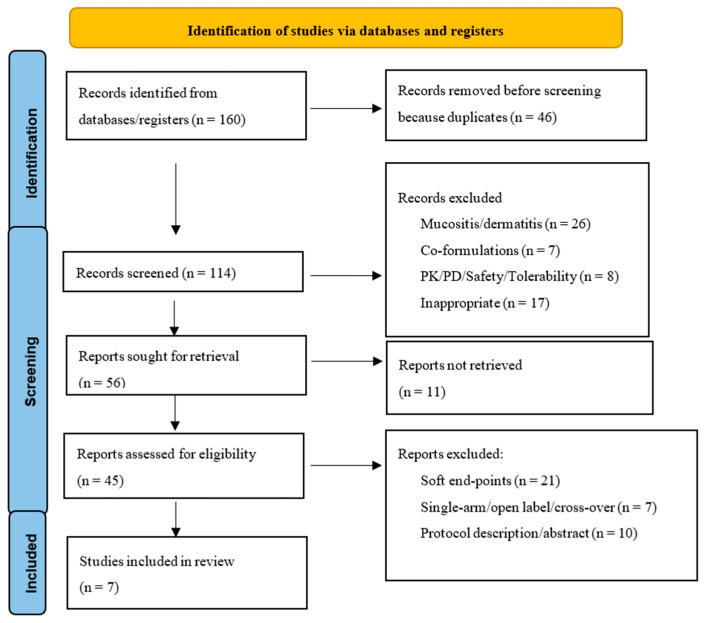
A flowchart of the methodology used to select RCTs according to PRISMA.

As shown in Figure 1, the literature search yielded 160 records. After removing duplicates (n = 46), 114 articles underwent screening based on title and abstract. Among these, 58 articles were excluded based on the criteria reported in the Section 2, leaving 56 articles whose full text was searched. Unfortunately, the full text of 11 articles was not found and only 45 articles underwent further analysis. Finally, based on the exclusion criteria, seven studies were selected and analyzed [28,29,30,31,32,33,34].

### 3.2. Study Characteristics and Synthesis

The included studies were published between 2016 and 2022 and evaluated the therapeutic use of curcumin in patients with different malignancies, including prostate (28.6%) [30,33], colorectal (28.6%) [28,32] and breast cancers (14.3%) [31], multiple myeloma (14.3%) [29], and oral leucoplakia (14.3%) [34]. The allocation ratio between the experimental arm and the control arm was 1:1, except for two studies with a 2:1 ratio [28,32]. Four out of seven articles (57.1%) were considered to have a low risk of bias [28,31,33,34], one (14.3%) was judged to have a high risk of bias [32], while two (28.6%) raised some concerns [29,30] (Table 2 and Table 3).

Most of the study populations considered in the included studies showed locally advanced or metastatic cancer and underwent other treatments (chemotherapy, radiotherapy) beyond the administration of curcumin, except for the studies by Kuriakose et al. [34] on patients with oral leukoplakia and Choi et al. [33] on patients with prostate cancers during the first androgen deprivation therapy withdrawal (Table 2).

In all studies, curcumin was administered orally, except in one study where the administration was intravenously [31]. The duration of the studies ranged between 16 weeks and 13 years (Table 2).

All the studies evaluated hard endpoints as primary outcomes, except for the study by Howells et al. [32], which only addressed the clinical benefit of curcumin in terms of OS and PFS as secondary outcomes (Table 2).

Cancer response, either clinical or objective/pathologic, was investigated as the primary endpoint in 57.1% of the studies [28,29,31,34]. Choi et al. [33] considered off-treatment duration, namely the time from the start of therapy withdrawal until progression, as the primary endpoint, while Passildas-Jahanmohan et al. [30] examined the time from inclusion to the first objective progression of the disease (Table 2).

The OS was considered in three studies (42.9%) [28,30,32]. On the whole, the OS did not show significant differences between arms except in the study by Howells et al. [32], which showed a better OS in the curcumin arm compared to the placebo (median time to death of 502 days and 200 days, respectively, in the per-protocol analysis). The PFS was assessed in five studies (71.4%) and did not show significant differences between patients receiving curcumin [28,30,31,32]. Lastly, regarding the cancer response, better results were detected in the curcumin arm compared to the placebo in three out of four studies (75%) [29,31,34], albeit the study by Gunther et al. [28] on colorectal cancer issued discordant results (Table 2).

The toxicity of curcumin was also taken into consideration in five studies (71.4%) [30,31,32,33,34]. Overall, few adverse events were reported in the curcumin arm even though the difference reached the significance in one study only [33] (Table 2).

## 4. Discussion

The results summarized in this systematic review, unfortunately, are not consistent enough to support the therapeutic use of curcumin, either as monotherapy or as an add-on to other standard antineoplastic drugs in patients with solid tumors (locally advanced or metastatic colorectal cancer, advanced metastatic breast cancer, metastatic prostatic cancer, and oral leukoplakia) or hematologic malignancies (multiple myeloma). Many factors may explain these inconclusive results, including the low number of clinical studies, the small number of subjects enrolled, the formulation and route of administration of curcumin, and the stage of the tumors studied (Table 2).

Among the seven RCTs analyzed, two studies were stopped early by the investigators because of the lack of any effect due to the small sample size, and one study was completed, although the researchers were aware that the number of patients enrolled was lower than that calculated in the protocol description [28,30,32,35]. Therefore, only four RCTs recruited the number of patients expected to achieve adequate statistical power [29,31,33,34]. Three out of these four RCTs were judged to have a low risk of bias (Table 3) [31,33,34]. Furthermore, some RCTs had several dropouts that further reduced the number of patients analyzed and the results obtained [29,31,32,33]. For these reasons, the conflicting results discussed below could be originally flawed by an inappropriate experimental design and under-sizing.

The heterogeneity of the curcumin formulations was another relevant factor. In two out of the seven RCTs analyzed, curcumin was given as the C3 complex [28,32]; in two others, curcumin was administered as the high bioavailable formulation BCM-95 CG [29,34], and three studies did not provide any detail on the curcumin formulation used [30,31,33]. The large dose range (the highest was 8 g/day for 6 weeks and the lowest was 1.44 g/day for 6 months) and erratic pharmacokinetic profile (Table 1) have made it difficult to compare the efficacy of these formulations. It is probably no coincidence that the only studies showing the beneficial effect of curcumin on cancer size or OS were those using high-dose BCM-95 CG (3.6 g/day for 6 months or 8 g/day for 28 days) [29,34]. Another approach to enhance absorption was to administer curcumin via the intravenous route because intravenous administration allows for bypassing the first-pass effect. Indeed, 300 mg of curcumin was intravenously administered once per week for 12 weeks as an add-on to paclitaxel, which induced a partial response in 50.7% of breast cancer patients treated with the taxane with respect to the placebo group [31]. Closely related to irregular absorption is the concern about the low levels of curcumin in tissues, in particular when considering that malignant cells uptake curcumin at a greater extent than normal ones [36]. In this regard, Gunther et al. [28] and Garcea et al. [37], who treated patients with colorectal tumors with the oral curcumin C3 complex (8 g/day for 6 weeks and 3.6 g/day for 1 week, respectively), found median curcumin concentration in the tumor tissues of 33.7 ng/mg and 12.7 ± 5.7 nmol/g tissue, respectively. Furthermore, in the rectal mucosa of healthy volunteers treated with the curcumin–phosphatidylcholine formulation, the mean level of curcumin was even lower, with a value of 2.8 ng/mL [13]. These concentrations, in the low nanomolar range, are several orders of magnitude lower than those used in cell-based studies to exert biological effects and may explain the diverse effects of curcumin in humans with respect to in vitro systems [28,38]. Unfortunately, no data are available about the concentration of curcumin in malignant tissues following the administration of BCM-95 CG or nanoparticle formulations, and this kind of study would be more than welcome. Taken together, these observations imply that the formulation and route of administration are key determinants of the therapeutic efficacy of curcumin.

Another issue worthy of discussion is the type of tumor and the stage of the disease. Five out of seven RCTs reviewed in this article were carried out in patients with metastatic colorectal, breast, or prostate cancer [28,30,31,32,33]. The lack of effect of curcumin in improving the OS, PFS, and TTP may be due to the advanced and disseminated stage of the tumors, which has rendered any proapoptotic, anti-inflammatory, and anti-angiogenic modification useless. This hypothesis is confirmed by the evidence that curcumin decreased proinflammatory cytokines and transcription factors (tumor necrosis factor, IL-6, NFkB, and VEGF) in multiple myeloma patients, as well as the tumor markers carcinoembryonic antigen and prostate-specific antigen in breast and prostate cancer patients, respectively [29,31,33]. As a whole, these findings suggest that the compound has been able to reach the targets, but these modifications were not strong enough to counteract tumor progression [31,33]. Studying the effects of curcumin in patients with early-stage tumors would give us a correct evaluation of its full therapeutic potential.

A working hypothesis that could justify the use of curcumin as an add-on to antiblastic agents is the ability of the former to inhibit drug-metabolizing enzymes. Curcumin has been reported to inhibit liver cytochrome P450 (CYP) isoform 3A4 (CYP3A4), glutathione-S-transferase (GST), and UDP-glucuronosyltransferase (UGT) and, by doing so, it could increase both the plasma and tissue levels of antineoplastic drugs, enhancing their therapeutic effect [2,39]. The clinical studies described above do not seem to support this theory. Indeed, docetaxel and paclitaxel both undergo liver metabolism through CYP3A4; therefore, if curcumin had inhibited this isozyme, one would have expected a potentiation of taxane anticancer effects in metastatic prostate and breast cancer patients but unfortunately, this has not occurred [30,31,40]. Actually, neither Passildas et al. [30] nor Saghatelyan et al. [31] measured the taxane plasma levels in curcumin-treated patients, and this possible drug–drug interaction was not adequately addressed. The use of oral curcumin for boosting anticancer drugs is an attractive approach and could open new avenues for alternative therapeutic use of curcumin in cancer.

A common outcome of these studies is the appreciable safety profile of curcumin. Cancer patients, treated with either oral curcumin (at doses up to 8 g/day for a maximum of 6 months) or intravenous curcumin (300 mg once a week for 12 weeks), experienced mild and transient adverse effects, including gastrointestinal discomfort (diarrhea, nausea, vomiting, and dyspepsia), hypertension, tachycardia, and anemia [30,31,32,33,34]. Interestingly, a reduced rate of lymphopenia and hypocalcemia were reported in breast or prostate cancer patients treated with curcumin [30,31].

The theme of drug–drug interactions, introduced above, can be a double-edged sword. Indeed, CYP3A4, GST, and UGT metabolize several drugs used by cancer patients for concomitant diseases, such as opioid analgesics (methadone, morphine), macrolide antibacterials (erythromycin, clarithromycin), azole antifungals (itraconazole, miconazole), corticosteroids (prednisone, prednisolone), etc. [40]. Therefore, curcumin, by inhibiting phase I and phase II enzymes, could increase the plasma levels of concomitant drugs giving rise to harmful side effects.

The results summarized in this systematic review, however, should be read bearing in mind the limitations of the work. As with all systematic reviews, a selection bias could not be completely ruled out, albeit a standardized approach was undertaken to perform the search and selection of eligible studies. Furthermore, as already addressed, included studies were deeply heterogeneous and showed several concerns that prevented making a quantitative synthesis and reaching a conclusive picture of the effect of curcumin on hard endpoints.

## 5. Conclusions and Future Directions

After a careful evaluation of the clinical evidence currently available, the conclusion that can be drawn is that curcumin is not an effective compound in either blocking or slowing down the progression of cancer. These conclusions, however, were drawn after a thorough analysis of only seven RCTs that evaluated hard endpoints, many of which were affected by some level of bias or undersized and/or had many dropouts that affected the final evaluation. Furthermore, the heterogeneity of the curcumin formulations, with their pharmacokinetic variability, and their use in patients with locally advanced or metastatic cancers, undoubtedly contributed to a possible underestimation of its potential clinical benefit.

Nevertheless, the acceptable safety profile and the evidence that curcumin may improve local symptoms of cancer-related radiodermatitis or mucositis bolster the interest of the scientific community to continue studying this compound [22,23,24,25]. In addition, the increased uptake of curcumin by tumor cells, along with the in vitro evidence that curcumin’s pro-apoptotic effect increases with the intracellular concentration, deserve more attention and should be taken into greater consideration for further development of novel formulations targeting cancer cells [36]. Finally, closer collaboration between basic researchers and oncologists would be desirable, not only to foster the exchange of expertise but also to enhance knowledge and accelerate the transition from preclinical to clinical research. The study of the efficacy of curcumin in early-stage tumors could also reserve pleasant surprises in a therapeutic sense.

## Figures and Tables

**Table 1:** Pharmacokinetic parameters of main curcumin formulations.

Formulation	Pharmacokinetic Parameters				Refs.
	AUC_0–24h_ (ng/mL h)	T_max_ (h)	C_max_ (ng/mL)	T_1/2_ (h)	
Curcumin C3 ^a^ (Sabinsa Corporation, East Windsor, NJ, USA)	731.6 ^e^	2–7	32–103 ^e^	-	[13,14]
BCM-95 CG ^b^ (Arjuna Natural Extracts, Ltd., Aluva, India)	3201.3 ^f^	3.5	456.88 ^f^	5	[15]
PC-curcumin ^c^ (Meriva, Indena S.p.A., Milan, Italy)	669.4 ^g^	0.5–2	42–119 ^g^	22.8 ± 34.2	[13,14,16]
NP-curcumin ^d^ (Theracurmin, Theravalues Corporation, Tokyo, Japan)	2649 ± 350 ^h^ 3649 ± 430 ^i^	1–6 ^h^ 2–6 ^i^	189 ± 48 ^h^ 275 ± 67 ^i^	9.7 ± 2.1 ^h^ 13 ± 3.3 ^i^	[17]

^a^ 73–80% curcumin, 20–27% demethoxycurcumin, and bisdemethoxycurcumin. ^b^ 95% curcuminoid complex (~95% curcumin; the remainder is demethoxycurcumin and bisdemethoxycurcumin) combined with turmeric essential oil enriched to sesquiterpenoids (see text). ^c^ 18–22% curcuminoids (curcumin, demethoxycurcumin, and bisdemethoxycurcumin in their natural ratios), phospholipids, and other compounds (see text). ^d^ 10% curcumin, 2% demethoxycurcumin and bisdemethoxycurcumin, and 88% other compounds (see text). ^e^ Dose: 4 g/day for 7 days. ^f^ Dose: 2 g/day, single dose. ^g^ Dose: 2 g/day for 7 days. ^h^ Dose: 150 mg, single dose. ^i^ Dose: 210 mg, single dose. AUC, area under the curve; C_max_: peak plasma concentration; NP, nanoparticles; PC, phosphatidylcholine; T_1/2_, half-life; T_max_, time to reach the peak plasma concentration.

**Table 2:** Summary of the descriptive characteristics of the RCTs included in the analysis.

First Author, Year	Country	Study Design	Duration of Study	Sample Size	Study Population	Age (Years Range)	Males (%)	Intervention	Study Endpoints (Time)	Experimental Arm N° (%)	Control Arm N° (%)	Hazard Ratio
Gunther et al. [28]	Germany	Phase II RCT	13 years	22 (E: 15;C: 7)	Patients with either T3/T4 or T2 and node-positive locally advanced CRC	28–75	59%	E: capecitabine (825 mg/m^2^ per os + RT (50.4 Gy in 28 fractions) + curcumin C3 complex (8 g/day per os during RT and for 6 weeks after its completion) C: capecitabine + RT as above + placebo	Pathologic complete response (at the time of surgery)	1 (7%)	2 (33%) *	
									Overall survival (5 years)	85.7%	85.7%	
									Progression-free survival (5 years)	66.7%	71.4%	
									Cumulative incidence of local regional failure (5 years)	6.7%	14.3%	
									Cumulative incidence of distant failure (5 years)	33.3%	28.6%	
Santosa et al. [29]	Indonesia	Pilot RCT	16 weeks	33 (E: 17; C: 16)	New patients with MM who were ineligible for transplant and were not previously treated	31–77	60.6%	E: MP regimen (melphalan 4 mg/m^2^ per os, prednisone 40 mg/m^2^ per os for 7 days) + curcumin (BCM-95 CG 8 g/day per os for 28 days) C: MP + placebo	Remission (4 months)	9 out of 12 patients who completed the follow-up (75%)	4 out of 12 patients who completed the follow-up (33.3%)	
Passildas-Jahanmohan et al. [30]	France	Phase II RCT	18 weeks	50 (44 in the ITT) [E: 26 (22); C: 24 (22)]	Patients with stage IV PC with documented castration resistance who were previously submitted to surgery, therapy, or hormonotherapy	44–87	100%	E: docetaxel (75 mg/m^2^ IV on day 1 every 3 weeks for 6 cycles) + prednisone/prednisolone (10 mg/day per os) + curcumin (6 g/day per os for 7 days every 3 weeks for 6 months) C: docetaxel and prednisone/prednisolone as above + placebo	Progression-free survival	5.3 months	3.7 months	N.A.
									Cumulative progression-free survival (6 months)	31.8%	45.5%	
									Overall survival	15.8 months	19.8 months	N.A.
									Cumulative survival (12 months) Cumulative survival (24 months)	60.1% 20%	80% 29.3%	
									Grade 3 or 4 adverse events	10	6	
Saghatelyan et al. [31]	Armenia	Phase II RCT	23 weeks	150 (E: 75; C: 75)	Patients with progressive, locally advanced, or MBC after at least one prior chemotherapy regimen who had progressed during or within 12 months of completing adjuvant or neoadjuvant chemotherapy or other cases of BC in which weekly paclitaxel was considered an adequate approach	28–75	0%	E: paclitaxel (80 mg/m^2^ IV once every 7 days for 12 weeks) + curcumin [(CUC-01) 300 mg IV once every 7 days for 12 weeks] C: paclitaxel as above + placebo	Objective response rate (16 weeks) Objective response rate (24 weeks)	38 (all PR, 50,7% ITT and 61.3% among 47 patients who completed the treatment) 22 [1 CoR and 21 PR, (29% in ITT, 44.9% among 47 patients who completed the treatment)]	25 (all PR, 33.3% in ITT and 38.5% among 46 patients who completed the treatment) 15 (all PR, 20% in ITT, 27.8 among 46 patients who completed the treatment)	
									Progression-free survival	27.0 weeks	24.6 weeks	1.28 (95% CI: 0.765–3.135)
									Stable disease (16 weeks) Stable disease (24 weeks)	18 (24% in ITT) 12 (16% in ITT)	26 (34.77% in ITT) 15 (20% in ITT)	
									Progressive disease	5 (6.7%)	14 (18.7%)	
									Patients with any adverse event	39 (54%)	42 (56%)	
									Patients with grade 3–4 adverse event	3 (4%)	2 (2.7%)	
Howells et al. [32]	United Kingdom	Phase IIa RCT	24 weeks	27 (E: 18; C: 9)	Patients with stage IV CRC without previous treatment	53–78	N.A.	E: FOLFOX ± bevacizumab (80 mg/m^2^ once every 7 days for 12 weeks) + curcumin C3 complex (2 g per os, once every 2 weeks for 12 cycles) C: FOLFOX ± bevacizumab (once every 2 weeks for ≤12 cycles or until patient progression, unacceptable toxicity, death, or withdrawal) + placebo	Overall survival (ITT)	N.A.	N.A.	0.339 (95% CI: 0.141; 0.815)
									Overall survival (PP)	596 days	200 days	0.271 (95% CI: 0.106; 0.697)
									Cumulative overall survival (PP) (6 months)	93.3%	55.6%	
									Progression-free survival (ITT)	N.A.	N.A.	0.571 (95% CI: 0.24; 1.36)
									Progression-free survival (PP)	320 days	171 days	0.549 (95% CI: 0.225; 1.34)
									Cumulative progression-free survival (PP) (6 months)	73.3%	33.3%	
									Objective response (6 cycles) Objective response (12 cycles)	66.7% 53.3%	44.4% 11.1%	
									Total adverse events	282	103	
Choi et al. [33]	South Korea	RCT	36 months	97 (E: 49; C: 48)	Patients with stage IV PC with BCR after localized treatments or metastatic PC at initial diagnosis who received LHRH agonist and anti-androgens for at least 6 months with a subsequent ADT withdrawal period	E (mean ± SD): 71.5 ± 9.0) C (mean ± SD): 72.9 ± 6.0)	100%	E: ADT withdrawal (deprivation after LHRH agonist and anti-androgens for at least 6 months) + curcumin (1440 mg/day per os, 2 capsules for 3 times/day for 6 months) C: ADT withdrawal (as above) + placebo	Off-treatment ^#^	16.3 months	18.5 months	N.A.
									PSA progression (6 months)	10.3%	30.2%	
									Patients who had adverse events	7 (15.6% out of 45 patients who ingested the test food more than once after randomization)	16 (34.8% out of 46 patients who ingested the test food more than once after randomization)	
Kuriakose, et al. [34]	India	Phase IIb RCT	12 months	223 (E: 111; C: 112)	Patients with clinical and histologically confirmed oral leukoplakia of a size more than 15 mm^2^ in area, with any linear dimension more than 1 cm, and without previous treatment	26–74	72.2%	E: curcumin BCM-95 CG (3.6 g/day per os for 6 months) C: placebo	Clinical response based on the lesion size (6 months)	75 (67.5% out of 105 available at the end of 6 months for the evaluation of primary endpoints)	62 (55.3% out of 108 available at the end of 6 months for the evaluation of primary endpoints)	
									Histologic response (6 months)	25 (22.32%)	23 (20.53%)	
									Combined clinical and histologic response (6 months)	65 (58%)	50 (44.64%)	
									Clinical response based on the lesion size (12 months)	29 (54.7% among 103 subjects with a 50% or greater decrease in the lesions at 6 months)	30 (60% among 103 subjects with a 50% or greater decrease in the lesions at 6 months)	
									Subjects experiencing any adverse events	26 (23.4%)	35 (31.3%)	
									Subjects experiencing moderate/severe adverse events	4 (3.6%)	18 (16.1%)	

ADT: androgen deprivation therapy; BC: breast cancer; BCR: biochemical recurrence; C: control arm; CoR: complete response; CRC: colorectal cancer; E: experimental arm; FOLFOX: Oxaliplatin 85 mg/m^2^ IV plus folinic acid 350 mg IV plus 5-FU 400 mg/m^2^ IV; ITT: intention to treat; IV: intravenously; LHRH: LH-releasing hormone; MBC: metastatic breast cancer; MM: multiple myeloma; N.A.: not available; PC: prostate cancer; PP: per-protocol; PR: partial response; PSA: prostate-specific antigen; RCT: randomized controlled trial; RT: radiotherapy. ^#^ Off-treatment is defined as the time from the start of ADT withdrawal until the patient progressed clinically or had a PSA above a predetermined threshold. * One patient did not undergo surgery.

**Table 3:** Quality assessment and risk of bias in the analyzed RCTs.

Articles	D1	D2	D3	D4	D5	O
Gunther et al. [28]	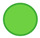	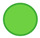	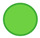	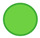	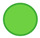	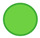
Santosa et al. [29]	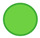	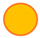	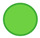	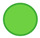	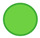	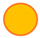
Passildas-Jahanmohan et al. [30]	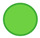	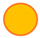	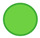	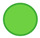	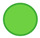	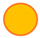
Saghatelyan et al. [31]	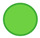	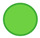	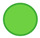	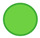	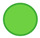	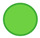
Howells et al. [32]	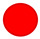	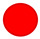	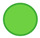	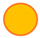	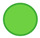	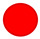
Choi et al. [33]	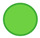	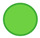	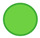	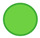	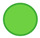	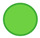
Kuriakose et al. [34]	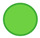	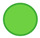	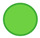	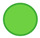	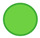	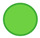

Domain (D) D1, randomization process; D2, deviations from intended interventions; D3, missing outcome data; D4, measurement of the outcome; D5, selection of the reported results; O, overall evaluation (for further information, see the Section 2). Red circles: high risk; green circles: low risk; yellow circles: some concerns.

## Data Availability

See Section 2.1.
